# 25th ANNIVERSARY OF CLONING BY SOMATIC-CELL NUCLEAR TRANSFER: Nuclear transfer and the development of genetically modified/gene edited livestock

**DOI:** 10.1530/REP-21-0078

**Published:** 2021-05-04

**Authors:** Ramiro Alberio, Eckhard Wolf

**Affiliations:** 1 School of Biosciences University of Nottingham, Nottingham, UK; 2 Gene Center and Department of Veterinary Sciences, LMU Munich, Munich, Germany

## Abstract

The birth and adult development of 'Dolly' the sheep, the first mammal produced by the transfer of a terminally differentiated cell nucleus into an egg, provided unequivocal evidence of nuclear equivalence among somatic cells. This ground-breaking experiment challenged a long-standing dogma of irreversible cellular differentiation that prevailed for over a century and enabled the development of methodologies for reversal of differentiation of somatic cells, also known as nuclear reprogramming. Thanks to this new paradigm, novel alternatives for regenerative medicine in humans, improved animal breeding in domestic animals and approaches to species conservation through reproductive methodologies have emerged. Combined with the incorporation of new tools for genetic modification, these novel techniques promise to (i) transform and accelerate our understanding of genetic diseases and the development of targeted therapies through creation of tailored animal models, (ii) provide safe animal cells, tissues and organs for xenotransplantation, (iii) contribute to the preservation of endangered species, and (iv) improve global food security whilst reducing the environmental impact of animal production. This review discusses recent advances that build on the conceptual legacy of nuclear transfer and – when combined with gene editing – will have transformative potential for medicine, biodiversity and sustainable agriculture. We conclude that the potential of these technologies depends on further fundamental and translational research directed at improving the efficiency and safety of these methods.

## What did Dolly teach us?

The germ-plasm theory by August Weismann proposed that cells of a developing organism lose developmental plasticity during differentiation (Weismann *et al.* 1889). Observations in the roundworm *Parascaris equorum* made by Theodor Boveri, showing chromosome diminution in the somatic compartment whilst a full chromosome set was retained in the germline, contributed to Weismann’s concept. Hans Spemann proposed that transferring the nucleus of a cell into an egg would be a 'fantastical experiment' that would put this idea to the test (Spemann 1938). Early experimental attempts in amphibians supported this idea, as embryonic development failed after the nuclear transfer of gastrula-derived somatic cells into oocytes (King & Briggs 1955). Furthermore, when primordial germ cells (PGCs) from the same stages were used as nuclear donors, normal tadpoles developed, implying that developmental plasticity was restricted in cells adopting a somatic identity (Smith 1965). However, subsequent work by using more advanced developmental stages, such as embryonic gut epithelial cells, resulted in the development of normal adult frogs after the nuclear transfer (NT). Although the efficiency was very low (~1%), this experiment offered the first evidence that the genetic content of differentiated cells was equivalent to that of an undifferentiated blastomere (Gurdon & Uehlinger 1966). However, adult frogs were never obtained after NT with adult cells, thus a demonstration of complete reprogramming of an adult somatic cell remained unanswered for another three decades. A report of four cloned cattle made from NT embryos reconstructed with cultured inner cell mass (ICM) cells suggested that partially differentiated donor cells supported full-term development in mammals (Sims & First 1994). The year 1996 saw the culmination of extensive efforts by many groups over previous decades in overcoming the technical challenges of performing NT in mammals. For the first time, cultured cells from established cell lines from the embryonic disc of a sheep embryo were successfully used as nuclear donors. These experiments resulted in the birth of two lambs, Megan and Morag, who grew to fertile adults (Campbell *et al.* 1996*b*). Following this experiment, the team used cells isolated from the mammary gland of a 6-year-old *Finn Dorset* sheep and performed 277 NT experiments, from which one lamb, *Dolly*, was born (Wilmut *et al.* 1997). A key insight for the success of these experiments was the understanding of the critical need for cell-cycle coordination between the donor cell and the recipient oocyte (Campbell & Alberio 2003). Previous work by Campbell and colleagues had established the importance of using cells in the non-replicative phase of the cell cycle for NT into metaphase II oocytes (Campbell *et al.* 1996*a*). This was achieved very ingeniously via the 'starvation' of cells for several days, in order to slow down cell-cycle progression and enrich for cells in G0. As a result, reconstructed embryos had normal ploidy. This was in contrast to embryonic blastomeres, which have a long S-phase and undergo DNA damage after transfer into an MII oocyte (Campbell *et al.* 1993). DNA damage contributes to widespread chromosomal abnormalities, consistent with those reported in early studies in amphibians.

The biological significance of Dolly the sheep, the first cloned mammal by using an adult somatic cell, was far-reaching. First, it answered the long-standing question of genetic/cellular equivalence among cells in the adult organism, which had occupied the minds of scientists for over a century. Secondly, it represented a new dawn for biotechnological applications in medicine and agriculture. Importantly, NT carried out under optimized conditions can erase epigenetic memory of somatic cells enabling multiple rounds of re-cloning without loss of developmental potential (Wakayama *et al.* 2013), emphasizing the powerful reprogramming capacity of oocytes (Alberio *et al.* 2006, Halley-Stott *et al.* 2013). Indeed, the physiological parameters of 6-year-old *Dolly* clones were equivalent to age-matched control animals, which indicates that NT does not have long-term detrimental effects on aging (Sinclair *et al.* 2016). Thus, although the overall efficiency of NT remains low, the animals that develop full-term can be clinically healthy and fertile.

## Genetic modification in livestock

Genetic modification of animals has primarily relied on the genetic modification of mouse embryonic stem cells (ESC) and the generation of chimeric founders that are bred to homozygosity (Doetschman *et al.* 1987, Thomas & Capecchi 1987). Mice have a short intergenerational interval and stem cell technologies have been available since 1981 (Evans & Kaufman 1981, Martin 1981). Hitherto, these methods have not been adopted in livestock because germline competent ESC were not available. However, two recent reports suggest that this may no longer be a limitation. They demonstrated two novel sources of embryonic stem cells derived from pig and horse pre-implantation embryos capable to robustly generate PGCs* in vitro* and can contribute to chimeric foetuses (Gao *et al.* 2019, Yu *et al.* 2020). The authors indicate that this technology can also work in other species, which would offer exciting opportunities for genetic engineering (GE) in livestock species. However, demonstration of efficient germline contribution in chimeric offspring is still needed before the broad use of livestock stem cells for GE can be realized. Thus far, however, NT has been a valuable alternative strategy for the generation of GE livestock. It has been used for multiple purposes, such as the generation of animal models of disease, the development of genetically multi-modified organ donor pigs for xenotransplantation, the production of nutraceuticals, preservation of endangered species, and as a platform technology for enhancing livestock genetic selection. Examples of these applications and some of the challenges and future directions are presented in the following sections.

## Tailored large animal models for human diseases

**Figure 1 fig1:**
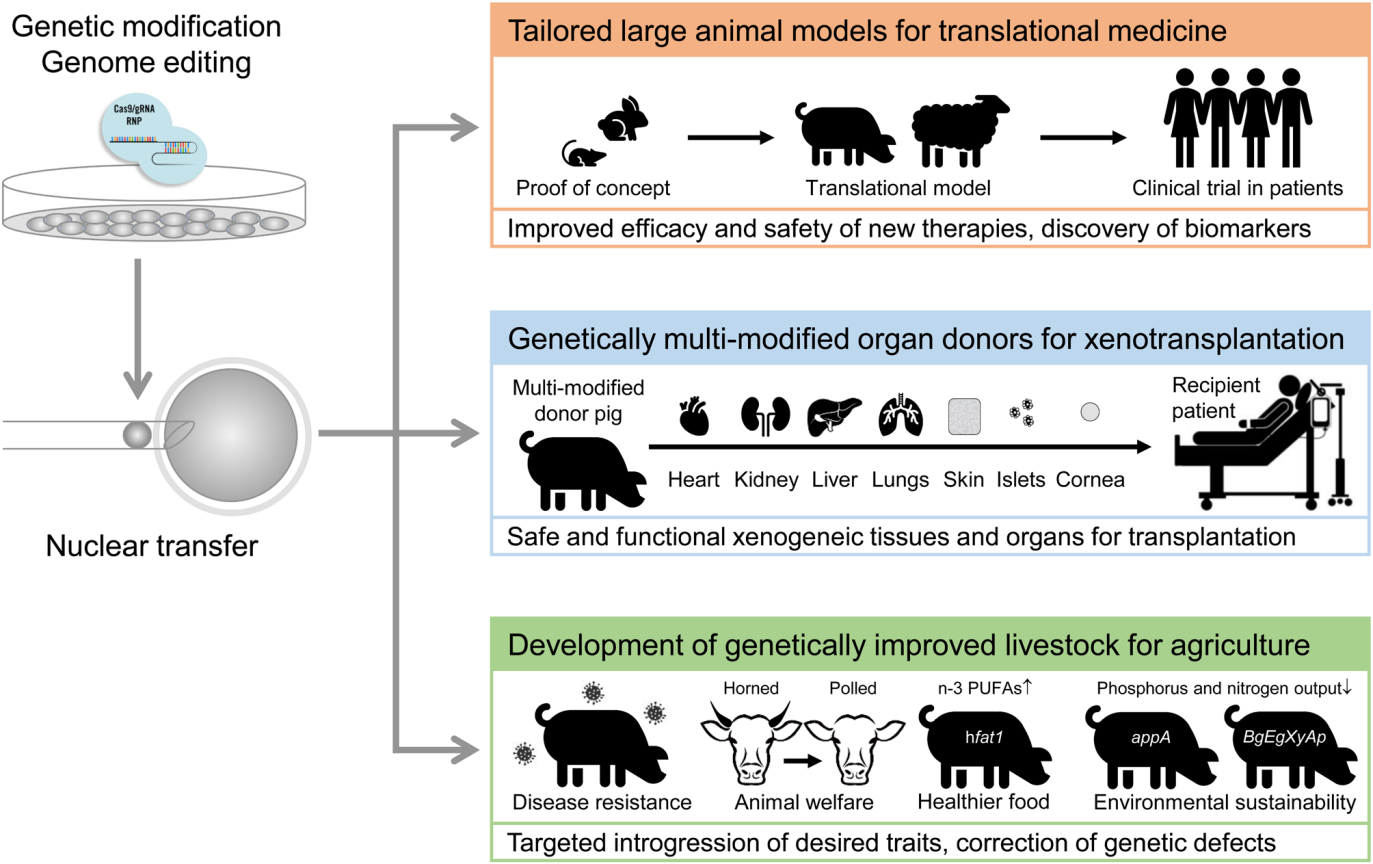
Nuclear transfer using genetically modified cells as technological pipeline for the generation of large animal models for translational medicine, organ donor pigs for xenotransplantation, and new developments for sustainable animal agriculture. PUFA, polyunsaturated fatty acid; h*fat1,* humanized version of the *C. elegans fat1* desaturase gene; *app,* expression cassette for microbial phytase; *BgEgXyA,* polycistronic expression cassette for beta-glucanase, xylanase, and phytase.

Livestock species share many anatomical and physiological characteristics with humans, such as large body size, similar metabolism and long lifespan, which are desirable when modelling human development and studying disease. Furthermore, livestock species are mostly outbred, making phenotypic observations more relevant to humans (Fig. 1). Among livestock, the pig stands out as the species of choice for human disease modelling due to similarities in organ anatomy, size and physiology. Genetically engineered pig models of cardiovascular disease (Schneider *et al.* 2020), diabetes (Renner *et al.* 2010, 2020), cystic fibrosis (Rogers *et al.* 2008, Bartlett *et al.* 2016, Caballero *et al.* 2021), several types of cancer (Perleberg *et al.* 2018), Duchenne muscular dystrophy (DMD) (Klymiuk *et al.* 2013, Moretti *et al.* 2020) and neurodegenerative disorders (such as Huntington’s disease and spinal muscular atrophy) have shown to closely recapitulate the physiopathology of these human diseases (Yang *et al.* 2010, Baxa *et al.* 2013, Holm *et al.* 2016). Importantly, these models are currently being used as platforms for developing new treatments and diagnostic tools (Renner *et al.* 2016, Regensburger *et al.* 2019, Moretti *et al.* 2020).

Notably, all these models have been generated via NT by using genetically modified somatic cells. The creation of gene-targeted animals by using somatic cells is very laborious, requires intensive cell screening, multiple rounds of NT, and results in only low numbers of viable healthy offspring, which explains the relatively small numbers of animals generated so far. However, the development of gene-editing techniques by using the CRISPR/Cas9 system promises to drastically increase the efficacy of gene modification in somatic cells as well as directly in zygotes, which would remove the need for NT. For example, a new DMD pig model that displayed robust disease phenotype has been created by zygotic injection of Cas9 mRNA and guide RNA (Yu *et al.* 2016). However, DNA repair following double-strand breaks (DSB) caused by CRISPR endonucleases in zygotes is primarily driven by non-homologous end-joining (NHEJ), which results in a high proportion of mosaic embryos. One way to reduce mosaicism has been to optimize the injection of the Cas9/gRNA complex during the first few hours (~5 h in pig and ~10 h in bovine), before the onset of the S-phase in the zygote, greatly reducing mosaicism (Park *et al.* 2017, Lamas-Toranzo *et al.* 2019). Another key aim of gene editing is the generation of a targeted knock-in via homologous recombination (HR). Since homology-directed repair (HDR) is not the preferred mechanism for DSB repair, methods that promote this process are needed. Complementation of Cas9/gRNA complex with RAD18, a component of the post-replicative repair pathway, increases HDR in cell lines, however, no data exist for embryos (Nambiar *et al.* 2019). The use of chemical compounds promoting HDR in bovine embryos has shown promising results yielding >50% gene targeting (Lamas-Toranzo *et al.* 2020). The simplicity of zygotic injection represents a major technological advantage over NT for the generation of gene-targeted animals and may replace the need of the latter in the future. It is also possible that microinjection may become less critical as methods for delivering one or multiple ribonucleoprotein complexes by electroporation are becoming more efficient in livestock (Tanihara *et al.* 2016, Hirata *et al.* 2020). A note of caution, however, needs to be made with regards to the high frequency of whole- or segmental-chromosome loss determined after DSB caused by the on- and off-target Cas9 cleavage during the zygote gene editing (Zuccaro *et al.* 2020). These observations call for the use of alternative approaches that do not require DSB to convert a targeted DNA into a new desired sequence, such as base or prime editors (Anzalone *et al.* 2020). Recently, transgenic chickens and pigs expressing Cas9 were reported as new resources for genome editing in livestock (Rieblinger *et al.* 2021).

## Genetically multi-modified donor pigs for xenotransplantation

The 'opt-out' system introduced as part of the changes to organ donation law in several countries was supposed to alleviate the waiting lists for organ transplantation. However, data from the UK shows that demand for organ transplants is growing at 1% per year, and the number of suitable donors is decreasing at a rate of 1–4% per annum. Reasons for the decline in available organs include older age of donors and obesity, both factors that contribute to adverse effects on transplant outcomes. The highest organ demand is for kidneys, pancreata, hearts and livers. The use of animal organs from livestock, such as pigs, offers a possible solution to this growing problem (Fig. 1). The pig has many advantages, including human-like size and physiology, broad availability and breeding characteristics (large litters and short reproductive cycles). Furthermore, pigs are amenable to advanced reproductive and genetic engineering platforms (reviewed in Kemter *et al.* 2020). Public acceptance for using pigs as organ sources is growing, although strict regulatory measures that ensure safe and ethically sustainable sourcing of organs are imperative (Kogel & Marckmann 2020). The technology has now reached a stage that offers hopes for clinical applications in the not-so-distant future.

Pig heart transplantation to non-human primates has been widely used over decades as a model system (Cooper *et al.* 2014). NT technology was used to produce multi-modified pigs devoid of alpha-1,3-galactosyltransferase (GGTA1), and expressing human complement regulatory protein CD46 and human thrombomodulin. Hearts from these triple-modified pigs survived for over 900 days after heterotopic abdominal transplantation in baboons (Mohiuddin *et al.* 2016). Remarkably, pig hearts with these genetic modifications showed consistent life-supporting function after orthotopic transplantation in baboons with survival times of up to 195 days (Langin *et al.* 2018, Reichart *et al.* 2020). Survival for a longer period was mainly limited by the continued growth of the pig hearts in the small chests of the recipient baboons. Inactivation of the growth hormone receptor gene (*GHR*) in the donor pigs is a potential strategy to overcome this problem (Hinrichs *et al.* 2021).

Besides the heart, multi-modified pigs (GGTA1-deficient/human *CD55* transgenic) made by NT have been used for kidney transplantation in a baboon that survived for 136 days (Iwase *et al.* 2017*a*, Iwase *et al.* 2017*b*). Moreover, transplantation of similar kidneys into macaques resulted in more than 1-year survival (Kim *et al.* 2009). For liver and lung xenotransplantation, the survival is more limited, however, the best results were obtained by using multi-modified pigs. Multiple other genetic modifications have been proposed to overcome humoral and cellular rejection of xenotransplants, to prevent coagulation disorders and other physiological incompatibilities, and to reduce/eliminate the risk of transmission of porcine endogenous retroviruses (Table 1; reviewed in Kemter *et al.* 2020).

**Table 1:** Genetic modifications of donor pigs of cells, tissues and organs for xenotransplantation.

Aim	Genetic modification
Deletion of sugar moieties of pig cells with pre-formed recipients’ antibodies	Knockout of the *GGTA1*, *CMAH*, *B4GALNT2* genes
Inhibition of complement activation	Transgenic expression of human complement regulatory proteins (hCD46, hCD55, hCD59)
Prevention of coagulation dysregulation	Transgenic expression of human THBD, EPCR, TFPI, ENTPD1, CD73 (NT5E)
Prevention of T-cell mediated rejection	Transgenic expression of human CTLA4-Ig, LEA29Y, PD-L1; knockout or knockdown of swine leukocyte antigens
Inhibition of natural killer cells	Transgenic expression of HLA-E/human β2-microglobulin
Inhibition of macrophages	Transgenic expression of human CD47
Prevention of inflammation	Transgenic expression of human TNFAIP3 (A20), HO-1, soluble TNFRI-Fc
Reduction of the risk of transmission of porcine endogenous retroviruses (PERV)	Knockdown of PERV expression; genome-wide knockout of the PERV *pol* gene

Recently, the combined use of CRISPR/Cas9 technology plus transposon-mediated transgenesis in somatic cells has been used to create NT-engineered pigs with multiple gene knockouts and human transgenes (Yue *et al.* 2020). The efficacy of these modifications was demonstrated in* in vitro* assays, but transplantation experiments in non-human primates still need to be performed. Newly created multi-modified pigs carrying eight human transgenes encoding for coagulation regulators, negative regulators of the immune response and complement system, plus the inactivation of three pig xeno-antigens were successfully used as donors of skin xenografts in *Cynomolgus* monkeys without the need of immunosuppressants for 25 days (Zou *et al.* 2020).

Thus, future milestones directed to reducing the size of the organs produced, compatible with humans, and increased tolerance through targeted gene modifications will pave the way to clinical assessment of these organs (Sykes & Sachs 2019, Reichart *et al.* 2020). The recent progress demonstrates the critical need for NT technologies for the generation of these donor animals for xenotransplantation that can now be enhanced by the incorporation of efficient gene-editing and -targeting technologies.

Another avenue that is being explored for autologous transplantation considers the creation of human organs in organogenesis-disabled pigs through interspecies chimerism. The proof of concept for this idea comes from a study showing the creation of a mouse with a rat pancreas following blastocyst complementation and interspecies chimerism (Kobayashi *et al.* 2010). Notably, in a reciprocal experiment, a mouse pancreas was created in a rat by blastocyst complementation. Isolated islets of Langerhans transplanted into a diabetic mouse restored normal glycaemia, demonstrating functional complementation (Yamaguchi *et al.* 2017). This experiment demonstrated the potential for creating functional organs using interspecies chimeras. As an alternative for human organogenesis, pigs and sheep are the desired host species. An initial study showed very limited contribution of human cells into pig chimeric foetuses following blastocyst complementation (Wu *et al.* 2017). The causes of the very limited chimerism are unclear, but they could be due to differences in the developmental stages represented by the stem cells and the host embryo (Mascetti & Pedersen 2016). The type of stem cell and the culture conditions can also determine the viability of the cells in interspecies chimeras (Fu *et al.* 2020, Aksoy *et al.* 2021). It is also noteworthy that the greater evolutionary divergence between pigs and humans (>90 million years) compared to that between mice and rats (<20 million years) renders this approach incompatible under these experimental conditions. However, a better understanding of the signalling pathways and pluripotency features operating in the early embryo could lead to developing better stage-matched complementation strategies. Comparative embryology using scRNASeq has revealed that conventional and naïve human cells are more closely matched to late pig blastocysts, suggesting that more advanced stages of pig development could be better hosts for human cells (Ramos-Ibeas *et al.* 2019).

The combination of strategies aiming at the 'humanization' of pig organs via (i) elimination of pig antigens, (ii) introduction of human immunomodulatory genes, and (iii) genetic ablation of pig organs followed by embryo complementation constitute avenues that are technically possible. Indeed, a recent study shows the generation of human–pig chimeric embryos using a combination of techniques. First, ablation of the *ETV2* gene, a master regulator of haemato-endothelium, was performed in porcine somatic cells. *ETV2* mutant embryos created by NT were then complemented with human-induced pluripotent stem cells (iPSC). Remarkably, the embryos generated contained blood vessels made exclusively of human endothelial cells (Das *et al.* 2020). The colonization of pig embryos was facilitated by the overexpression of *BCL2*, an anti-apoptotic gene. Previous work showed that inhibition of *BCL2* can overcome the staged-related barriers to colonization in chimeric embryos (Masaki *et al.* 2016). More recently, a new approach for increasing interspecies chimerism was reported, based on the creation of *Igf1r* mutant mouse embryos complemented with WT rat ESC (Nishimura *et al.* 2021). This resulted in the generation of neonatal mice having the extensive contribution of rat cells in diverse organs, predominantly those in which IGF signalling is very important, including the kidney, heart, lung and thymus. Although future studies should focus on the phenotypic analysis of such animals, this work shows the prospect of using pigs for creating organs containing autologous human vasculature, thus greatly reducing the chances of immune rejection.

## The impact of gene editing in animal production and welfare

The challenges of improving sustainable animal production whilst meeting the increased demand for healthier and nutritious animal products by the growing world population demand the use of new approaches to enhance the quality and productivity of livestock (Fig. 1). One approach suggested to improve the health benefits of meat consumption is to modify the proportion of unsaturated fats. Since dietary interventions to feed animals with sources of such fats are not environmentally friendly, transgenic approaches could be used. Examples of cattle, sheep and pigs expressing the *C. elegans fat1* desaturase gene in fibroblasts prior to NT have been reported (Lai *et al.* 2006, Wu *et al.* 2012, Zhang *et al.* 2013). These animals had a richer content in omega-3 fatty acids, making them a prime example of a nutraceutical produced by NT. Other examples include the generation of hypoallergenic milk through the abolition of β-lactoglobulin production in cows (Jabed *et al.* 2012), and lactoferrin and lysozyme-containing cow milk (Kaiser *et al.* 2017). However, current regulations for the consumption of products from genetically modified animals are very restrictive.

Another strategy for improving specific traits not reliant on transgenesis is to incorporate specific mutations that can alter animal phenotypes. For example, the naturally occurring mutation at the *MSTN* gene is the cause of the 'double muscle' phenotype in some cattle (Grobet *et al.* 1997, McPherron & Lee 1997) and sheep (Clop *et al.* 2006) breeds, resulting in 20% more muscle mass. These mutations were introduced not only into other sheep and cattle breeds (Proudfoot *et al.* 2015) but also into pigs (Wang *et al.* 2015*a*) and goats (Wang *et al.* 2015*b*) by gene editing via NT or zygotic injections. Gene editing offers an alternative means for the accelerated introduction of naturally occurring alleles, albeit in different species. From a regulatory and consumer perspective, the acceptability of such products may be less controversial.

Importantly, herd productivity is also dependant on the robust health status of the animals reared. Gene editing can be used to generate disease-resistant animals. Porcine reproductive and respiratory syndrome virus (PRRSV)-resistant pigs were created following the mutation of *CD163*, which prevents viral infection (Whitworth *et al.* 2016, Burkard *et al.* 2017). Similarly, transmissible gastroenteritis virus (TGEV)-resistant pigs were created by editing the gene for the putative viral receptor ANPEP(Whitworth *et al.* 2019). Other strategies include the use of gene introgressions to create disease-resistant pigs. The *RELA* gene from the warthog, naturally resistant to African swine fever, was introgressed into domestic pigs (Lillico *et al.* 2016). Although a delayed onset of infection was determined, this introgression did not confer complete protection against the clinical symptoms, suggesting that additional modifications may be needed (McCleary *et al.* 2020). Another recent study reports the use of a CRISPR/Cas9 nickase strategy combined with NT to generate cattle with a targeted insertion of a natural resistance-associated macrophage protein-1 (NRAMP1) expression cassette making them resistant to tuberculosis (Gao *et al.* 2017).

Part of improving production systems will require changes in the ways in which animals are reared. A good example of a step towards improving welfare is the propagation of the *POLLED* genotype across cattle breeds. By using gene editing and NT, *POLLED* alleles were introgressed and resulted in the birth of homozygous polled bulls (Carlson *et al.* 2016), which after crossing with horned cows delivered hornless offspring (Young *et al.* 2020).

Probably the most important goal of future animal production is environmental sustainability. Genetic engineering was thus used to overcome inefficient feed digestion in pigs, which results in excessive release of phosphorus and nitrogen to the environment. Transgenic pigs expressing microbial phytase in the salivary glands had an increased ability to digest phosphorus from dietary phytate and showed a markedly reduced faecal phosphorus concentration (Golovan *et al.* 2001). Recently, this approach was extended to transgenic pigs expressing beta-glucanase, xylanase, and phytase in the salivary glands. As a consequence, digestion of non-starch polysaccharides and phytate was enhanced, faecal nitrogen and phosphorus outputs were reduced (23–45%), and growth rate (~25%) and feed conversion rate (12–15%) were significantly improved (Zhang *et al.* 2018).

These examples demonstrate that favourable traits (e.g. disease resistance and feed conversion efficiency) can be rapidly introduced to improve sustainable production, health and welfare of animal production systems.

## Advanced animal breeding and genetic selection

The long generation intervals in domestic animals hinder the progress of genomic selection requiring novel approaches to accelerate the pace at which new animal phenotypes can be created. As discussed above, the combination of robust and safe gene/base-editing methods and reproductive techniques, such as* in vitro* fertilization and NT can drastically accelerate the rate of genetic gain. However, the bottleneck of meiosis remains. This critical step in the reproductive cycle of animals ensures genetic diversity, however, in breeding programmes, it represents a major hurdle due to the long time needed to generate mature gametes in livestock. Breeders and geneticists have been working on technological solutions to shorten this period for decades. A vision for utilizing* in vitro* systems for growing gametes as a means to reduce generation intervals, also known as 'velogenetics', was proposed in the early ’90s when genotype databases and assisted reproduction were beginning to be used (Georges & Massey 1991). More recently, these ideas have resurfaced as a result of developments in genetic selection, stem cell technologies and the possibilities of* in vitro* gamete production in domestic animals (Rexroad *et al.* 2019). *In vitro* breeding (IVB) was proposed as a platform combining the use of quantitative trait loci (QTL) datasets and reproductive techniques as a method of enhanced genetic selection (Goszczynski *et al.* 2019). This approach would yield a ten-fold increase in genetic selection without genetic manipulation. The use of gene/base-editing methods could further enhance the rate of genetic gain of this platform by multiplexing the incorporation of new alleles (Jenko *et al.* 2015).

However, this technology is contingent on a complete* in vitro* system for making gametes. Recent advances in our understanding of gamete development in large mammals are paving the way for the generation of mature gametes (Kobayashi *et al.* 2017). Remarkably, there are close similarities in the transcriptional programme between human, pig and cattle germline development (Soto & Ross 2021, Zhu *et al.* 2021), which offers advantages when it comes to translating findings from one species to another. This is particularly important because progress in human germ cell differentiation shows that mature oogonia can be generated, albeit at very low efficiency (Yamashiro *et al.* 2018). Detailed molecular understanding of the biology of germ cells has also enabled the creation of oocyte-like cells directly from mouse embryonic stem cells by the expression of eight specific transcription factors (Hamazaki *et al.* 2021). These oocyte-like cells were capable of chromosome segregation but failed to undergo normal meiosis. Nevertheless, these advances offer exciting opportunities for adapting some of these conditions for the generation of animal gametes from novel stem cells.

Considering the challenges of accomplishing meiosis* in vitro*, the technology of surrogate sires could serve as an alternative. The idea is to generate males that lack their own spermatogonia, but their testis can act as a developmental niche for spermatogonia from other males. Testis of *NANOS2* KO males support allogeneic spermatogonial transplantation and full spermatogenesis (Ciccarelli *et al.* 2020). The use of this technology could not only significantly increase the genetic merit of sires used in breeding programs (Gottardo *et al.* 2019) but can also serve as a tool for the genetic preservation of endangered species.

## Preservation of endangered species

Nuclear transfer offers an avenue for the rescue of endangered species, provided a compatible cytoplast can be obtained from closely related species. Some successes were reported with wild African cats and grey wolfs, but limited success was reported with an extinct subspecies of goat (Gomez *et al.* 2004, Kim *et al.* 2007, Folch *et al.* 2009). A critical aspect of this approach is the reliance on compatible cytoplasts. Importantly, a major breakthrough in the generation of synthetic cytoplasts from embryonic stem cells was recently reported (Hamazaki *et al.* 2021). Future experiments will determine whether this technology can be used as an alternative source of compatible cytoplasts suitable for nuclear transfer. This platform would offer a reproductive alternative for rescuing species such as the Northern White rhinoceros (Hildebrandt *et al.* 2018). In this case,* in vitro* fertilized embryos and embryonic stem cells have been produced from the last two female specimens of the species. The generation of gametes from these stem cells as well as the generation of cytoplasts would enable the propagation of these last remaining animals by both natural reproduction and nuclear transfer. Although a limited gene pool could represent an obstacle for expanding endangered species, the establishment of induced pluripotent stem cells from frozen tissues/blood could offer an alternative route for the generation of gametes that could be used for* in vitro* breeding.[Fig fig1]

## Concluding remarks

In this review, we summarized the critical impact that nuclear transfer had in facilitating the generation of genetically modified animals over the past 25 years. The impact of this technology has undoubtedly been more significant in livestock species due to the lack of embryonic stem cells, the standard route of gene targeting in mice. Thus, gene modification of donor cells prior to NT has been the preferred avenue for creating genetically modified livestock. Although the technique remains quite labour intensive, significant progress has been made in the procedures resulting in better efficiency. New sources of livestock embryonic stem cells have been reported, suggesting that gene manipulation and chimera generation may be simplified in the future. However, the long generation interval of livestock species makes the process of breeding to homozygosity of chimeric animals a lengthy and expensive process compared to mice. In contrast, NT allows the instant generation of transgenic animals, suggesting that it will remain a valuable technology for years to come.

We have come a long way since this landmark experiment, with improved knowledge of the molecular mechanisms of cellular reprogramming and more efficient techniques of NT. We can look forward to the next 25 years in which NT, in combination with other modern gene manipulation technologies, can offer solutions to urgent biomedical needs, improve sustainable animal production and facilitate species conservation.

## Declaration of interest

The authors declare that there is no conflict of interest that could be perceived as prejudicing the impartiality of this review.

## Funding

This work was supported by the Biotechnology and Biological Sciences Research Council (grant number BB/S000178/1) to R A and by the German Research Council (DFG; TRR127) and the German Center for Diabetes Research (DZD; 82DZD00802) to E W.

## Author contribution statement

RA and EW performed literature research and wrote the manuscript. EW prepared the figure and table. Both authors reviewed and approved the final version of the article.
